# SpatialAquaCrop, an R Package for Raster-Based Implementation of the AquaCrop Model

**DOI:** 10.3390/plants11212907

**Published:** 2022-10-29

**Authors:** Vinicius Deganutti De Barros, István Waltner, Rakotoarivony A. Minoarimanana, Gábor Halupka, Renáta Sándor, Dana Kaldybayeva, Györgyi Gelybó

**Affiliations:** 1 Doctoral School of Environmental Sciences, Hungarian University of Agriculture and Life Sciences, 2100 Gödöllő, Hungary; 2 Department of Water Management and Climate Adaption, Institute of Environmental Sciences, Hungarian University of Agriculture and Life Sciences, 2100 Gödöllő, Hungary; 3 Department of Geography, Oklahoma State University, Stillwater, OK 74078, USA; 4 Agricultural Institute, Centre for Agriculural Research, Eötvös Loránd Research Network, Martonvásár, Brunszvik u. 2., 2462 Martonvásár, Hungary

**Keywords:** AquaCrop, soil moisture, NDVI, biomass, crop cover, spatial, Hungary

## Abstract

Modeling crop water use and soil moisture availability is becoming increasingly critical, particularly in light of recent drought events. Our study focuses on the spatial application of the AquaCrop model, using a raster-based approach in an R-based environment. The formulated methodology was initially applied and tested on two point-based examples in the Central region of Hungary, followed by the spatial application of the model at the Rákos Stream catchment in the same region. For evaluation purposes, we also utilized satellite-based NDVI data. The results showed that there is a strong correlation between NDVI values and the model-based biomass estimation. We also found that the model simulated the soil moisture content fairly well, with a correlation coefficient of 0.82. While our results support the validity of the applied methodology, it is also clear that input data availability and quality are still critical issues in spatial application of the AquaCrop model.

## 1. Introduction

Production from agriculture has greatly increased in the past century, mostly due to productivity-enhancing technologies and an increase in efficiency in using natural resources for the purpose of enhancing agricultural production, especially for food [1]. Good and efficient management of natural resources still needs to be one of the goals of modern agriculture, especially when taking into consideration the necessity of preserving the natural environment [2]. 

As water management has become a critical issue for human well-being [3], for agriculture (crop production), different economic activities, and sustainable development [4], proper management is required, having agricultural sector sustainability as an aim [1]. Water is a renewable resource; however, due to increase in human population, water demands have continuously increased, and the uneven distribution of water availability makes it even harder to meet this demand [3,5]. Trends in terrestrial water storage indicate that freshwater resources are significantly affected by climate change and are overutilized in many regions [6], while water use has risen sixfold over the past 100 years [7]. Using AQUASTAT, a study confirmed that the growth rate of water use is about 1% per year [8], while Burek et al. [9] showed that by 2050, water use will further increase by 20–30%. Water issues have thus become among the top risks in the coming decade and have become one of the most important of the Sustainable Development Goals [3,6], leading to limits to economic development and security risks [6].

A cost-efficient way to test the response of agricultural systems to external changes without real-time observations (monitoring) is the use of crop models. Such models have been widely used and are receiving increased interest with improving data availability, while their improvement, application, and calibration are still the focus of multiple studies [10,11]. AquaCrop, a model developed by the Food and Agriculture Organization of the United Nations, is one of several different models that aid water management related to crop production. AquaCrop has been widely used for the calculation of evapotranspiration due to its strong equilibrium between robustness and precision [12]. AquaCrop is a general model for a large range of agricultural production (herbaceous, forage, vegetable, grain, fruit, oil, root, and tuber crops). In general, AquaCrop has been used to simulate crop development, yield production, and water-related variables such as evapotranspiration and water productivity, while considering different stress conditions [13] such as leaf growth and canopy expansion, stomatal conductance and canopy senescence, and pollination failure [13]. Different studies around the world have been conducted using AquaCrop, with some examples being a simulation of the water footprint of rice and maize in China [14], a calculation of irrigation technologies’ impact on cotton [15], and an evaluation of tomato’s water needs in Italy [16]. Rakotoarivony et al. [17] applied the AquaCrop model on a raster dataset using an R-based approach to determine spatial variations in seasonal evapotranspiration of maize.

Soil moisture is a key component of the hydrologic cycle, which is extremely variable and nonlinear in space and time [18], and it integrates interactions between the land surface and atmospheric processes [19]. Undoubtedly, soil moisture influences how an ecosystem responds to its physical surroundings by influencing the surface energy budget and the partitioning of rainfall into runoff or infiltration [20]. In recent decades, global climate change and human activities (such as overgrazing, mining, and water overuse) have had a significant influence on terrestrial ecosystems, significantly affecting rangeland ecosystems by altering land use/cover patterns and ecosystem water balances [8,19,21,22,23]. Soil moisture is influenced by different spatiotemporal variables such as diversity of altitude, topography, climate, and human interactions [24]. Besides these variables, there are others which can also affect soil moisture and are usually the ones taken into consideration for profile-based one-dimensional modeling: soil properties and vegetation cover [25].

Nowadays, given the increasing amount of available spatial and remotely sensed data, combined with the need for agricultural water management, an increased number of applications require raster-based utilization of crop models. The purpose of the current study is to demonstrate such a methodology. In data-scarce regions where accurate yield and soil moisture measurements are not available, we can utilize remote-sensing-based and model-based estimations. We developed a method that can be implemented on the spatial level but can also be continuously improved based on reliable surface measurements (ground truth). For this purpose, an R [26] based methodology [27] was developed to feed raster-based data to AquaCrop and to ultimately generate a raster output for water- and crop-related variables.

## 2. Materials and Methods

### 2.1. Experimental Data

To support this analysis and the usage of the developed R methodology [27], two different sites—one in Martonvásár, Hungary (47°18′18″ N, 18°48′49″ E) and the other one an experimental research field for MATE university in Gödöllő, Hungary (47°35′41″ N, 19°22′10″ E)—were used to validate the package results. For Martonvasar, values of maize soil moisture were used for validation, and for the experimental field in Gödöllő, different NDVI time series for the region were used for comparison with the modeled biomass data and green canopy crop cover (CC). It is known from many studies how well the AquaCrop model behaves, and it has been extensively validated [14,15,16,17], but for this paper, the points that were analyzed were to show the importance of having the results given by AquaCrop in a spatial manner.

**Figure 1:**
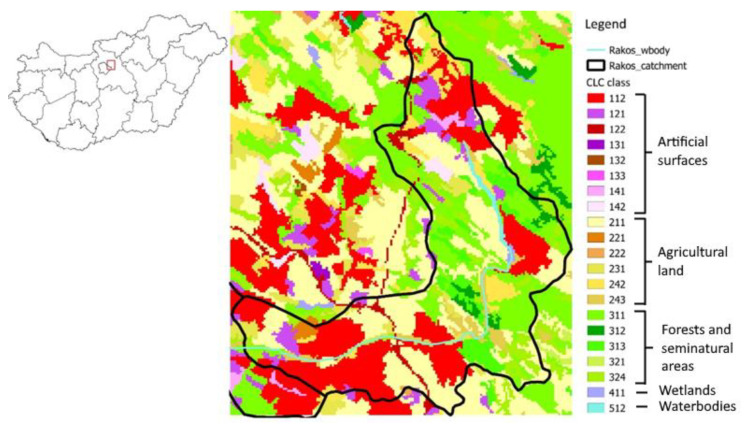
Land cover of the area based on the 2018 CORINE Land Cover dataset [28].

The primary focus of this study was the catchment area of the Rákos Stream and its surrounding region, which contains the experimental field in Gödöllő. The area is situated in the central region of Hungary, just east of the capital city of Budapest. The stream itself is 44 km long, with a 187 km^2^ catchment area, flowing from the Gödöllő Hills southward and then turning west to flow into the Danube River. As the lower 22 km of the stream flows through Budapest, where its catchment area is heavily urbanized, the current study only focused on the upper section of the basin. Figure 1 presents the land cover of the area based on the 2018 CORINE Land Cover dataset [28]. In 2018, about 32% of the area was covered by artificial surfaces (such as urban or industrial areas), 35% was covered by agricultural land (including pastures), 31% was covered by forests and semi-natural habitats (including natural grasslands), and 1% was covered by wetlands [29].

**Figure 2:**
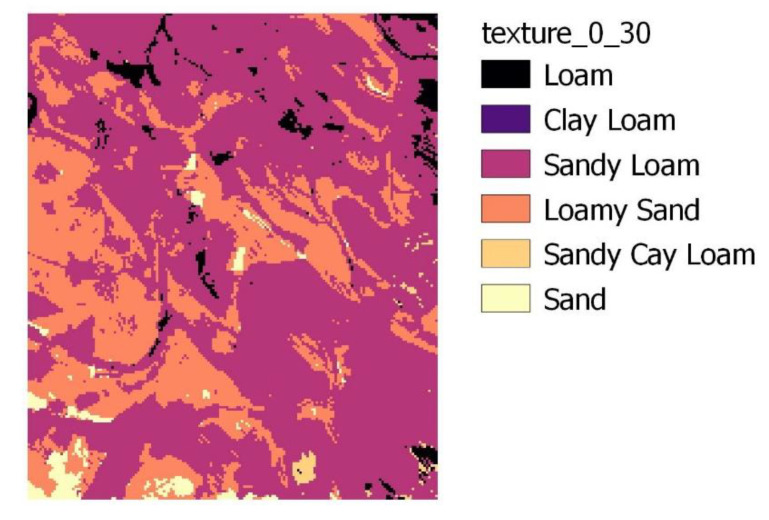
Texture map up to 30 cm soil depth, based on DOSoReMI.hu data [30].

For the Gödöllő site, soil data were accessed from two sources. Field capacity (FC), saturation (SAT), and permanent wilting point (PWP) data were downloaded from EU-SoilHydroGrids ver1.0 [30] (https://eusoilhydrogrids.rissac.hu/, accessed on 13 July 2022) with 250 × 250 m spatial resolution, while soil texture data were derived from the DOSoReMI.hu initiative [22] (with 100 × 100 m spatial resolution). Figure 2 presents the spatial variability of the soil texture of the top 30 cm layer within the study area. For the Gödöllő site, winter wheat was applied for both observed years (sowing dates: 1 December 2019 and 23 November 2020; harvest: 23 July 2020 and 26 July 2021).

The climate data (daily precipitation, maximum and minimum temperatures) used in the simulation for the entire catchment were accessed from the Meteorological Database of the Hungarian Meteorological Service (OMSZ) (https://odp.met.hu/, accessed 6 July 2022). Daily potential evapotranspiration was calculated using the Pennman–Monteith equation [31]. Climatic data were available at a 0.1° × 0.1° spatial resolution and were interpolated and resampled to the target 100 × 100 m grid.

For the other parameters, standard values (which are provided by FAO) were considered. As neither of the modelled crops (wheat and maize) are typically irrigated in the region, irrigation was not applied; only rainfed conditions were considered.

As for the validation at the Martonvasar site, the necessary soil and meteorological data for running the AquaCrop model were provided by the work of Sándor [32]. The maize field trial was established in 2020 at Martonvásár under ploughing and minimal tillage managements, aiming at the effect of cover crops sown for the winter period. The plot size was 35 m × 17.5 m for each treatment. The treatments were set up in two replicates. The maize (*Zea mais* L.) used on the field of the trial was sowed under conventional ploughing without a cover crop (i.e., the control treatment of the trial) as it represents the most typical management in the region. The chernozem soil of the experiment is nonacidic loam with a deep A horizon and 1.96–2.26 m% humus content. Maize was sown on the 16th of April and harvested on the 21st of October. Soil parameters for the model were obtained using field data on the soil physical properties and water retention. For the topsoil (0–30 cm), the texture was silt loam (FAO), with SWC = 49%, FC = 31%, PWP = 9%, and Ks = 9 cm/d.

For biomass and CC comparison, locally analyzed soil parameters and meteorological data from the local meteorological station (situated at the experimental field of the Hungarian University of Agriculture and Life Sciences, Gödöllő, Hungary) were utilized (precipitation, temperature), and from these, the reference evapotranspiration was derived utilizing the Pennman–Monteith equation [31].

Crop parameters for winter wheat and maize (for the Martonvásár site) were mostly kept the same as the standard ones provided in the AquaCrop software (version 6.1, FAO Land and Water Division, https://www.fao.org/aquacrop/en/, accessed 12 January 2021) (for the modeling). However, the number of days between sowing and emergence, maximum rooting depth, senescence, flowering, and maturity were changed in accordance with Szász [33], and winter wheat at the site (Gödöllő) was sown on the 1st of December and harvested on the 23^rd^ of July.

### 2.2. SpatialAquaCrop Overview

SpatialAquaCrop [27] is the name that was given to the developed R-based methodology. It was designed to be a user-friendly method in R to read spatial datasets and utilize the AquaCrop plug-in to run the AquaCrop model and then output the results as raster files. In the current version of its script, it can read TIF and netcdf files, and it can output the results as a TIF file. The output can be from a specific date or for the whole period of the run. The script provides functions to prepare initial model conditions and to run the AquaCrop plugin on a raster dataset.

The primary approach of the package is to run the external AquaCrop plugin software for each raster cell in the dataset by automatically generating the required input text files (based on user input data), running the model, reading the generated output files, and combining them into a raster output.

To run the AquaCrop plugin, the model requires weather data (precipitation, maximum and minimum temperatures, reference evapotranspiration, and atmospheric CO2 concentration), crop data, field management, irrigation, groundwater table, some initial conditions, if present, and the off-season conditions, if applicable. All of these must be present in special text files which the AquaCrop plug-in can read.

**Figure 3:**
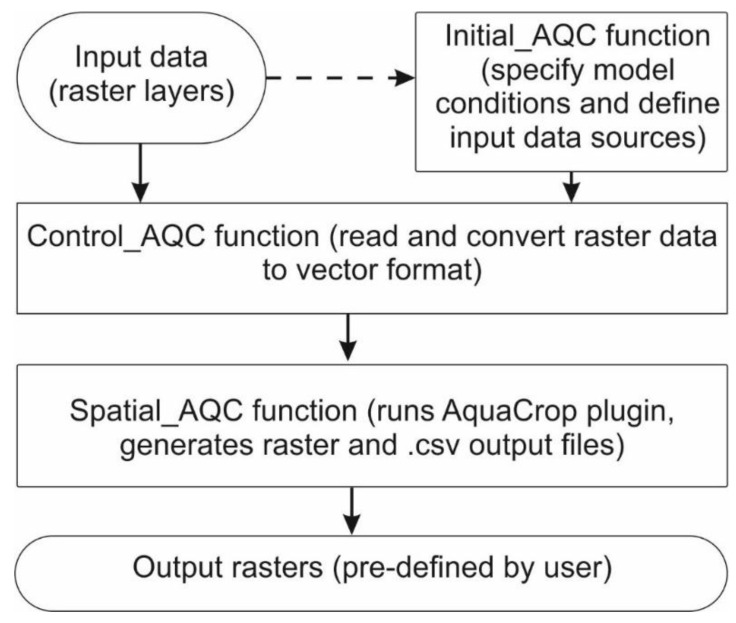
Workflow of the SpatialAquaCrop application.

Therefore, as of now, there are three scripts which represent the three main functions that aim to gather all the necessary information for the AquaCrop plug-in to run and output them in a raster format, with all of this done in a user-friendly way. Figure 3 presents the application workflow, while Table 1 shows an overall view of each of the functions.

The initial function is called ‘Initial_AQC()’. It aims to give the user the option to choose which inputs will be used for the model to run; as field management, irrigation, and groundwater tables do not require specific data inputs, the model can run utilizing some standard values for them. However, should specific information, such as irrigation data, be available, the data can be provided similarly to the standard irrigation file of the Aqua-Crop software. The function also provides a table to contain the paths of the different input files.

The file preparation function is called ‘Control_AQC()’. This second function aims to create the AquaCrop input files and other ones necessary for the main function.

The main function is called ‘Spatial_AQC()’. This last function aims to run all the spatial input data and output different raster files of some of the outputs that the AquaCrop model can provide (crop yield, evapotranspiration, runoff, drainage, biomass, and irrigation needed).

The scripts for this methodology are currently available at github [27], and in the future, the goal is to transform them into a functioning package for R. Initial_AQC uses the svDialogues [34] package to give different choices to the user on which optional inputs they would want to use for the model run; after this, different .csv files are created depending on the user’s choices. The second and third functions are more related to how they read the raster files and process them into the text files that are needed for the AquaCrop plugin to run. These functions utilize primarily the raster [35] and ncdf4 [36] packages.

### 2.3. General Methodology

The main goal of this research was to show the application of the AquaCrop model in a raster format applied within the R environment. First, a point-based validation was carried out for soil moisture for a maize field in Martonvasar for 2020. We can gain information on the performance of the model using the default maize crop file, as site-specific data are scarce in the area. Besides this validation, a comparison of the modelled biomass and green canopy crop cover against NDVI was done for winter wheat at an experimental site in Gödöllő for the years of 2020 and 2021; this comparison was done for the growth period until around senescence. As field-scale yield information is considered to be sensitive data, it is difficult to obtain from farmers; hence, we used NDVI as a proxy for biomass in the validation years. Following the validation efforts, the developed R methodology [27] was used to simulate wheat growth for the year of 2020 in the Rákos watershed region.

### 2.4. Point-Based Evaluation

Point-based evaluation of the AquaCrop model was carried out in two different sites: on a maize field in Martonvásár, regarding the surface soil moisture, and in the experimental field in Gödöllő (located inside the Rákos stream catchment), where an NDVI comparison between the modeled biomass and green canopy crop cover (CC) for winter wheat was made. Specific soil, climate, and crop data were taken in consideration for each of the sites for better parametrization of the model.

First, the NDVI index was calculated via Equation (1).

(1) $$NDVI=\frac{NIR-RED}{NIR+RED}\;$$

For this comparison, Sentinel 2 images were used to calculate the NDVI in specific band 8 for NIR (near-infrared) and band 4 for RED (red).

NDVI-based biomass values were calculated using Equation (2) [37].

(2) $$Biomass=10.728\times NDVI^{1.4315}$$

NDVI-based canopy cover (CC%) was calculated using Equation (3) [38]:

(3) $$CC\%=\frac{NDVI-NDVI_{soil}}{NDVI_{veg}-NDVI_{soil}}$$

where NDVI_soil_ is the NDVI value of the bare soil assumed on the sowing date (0.08 in the present study), while NDVI_veg_ is the NDVI value of a pure vegetation pixel (assumed to be 0.6 for wheat in this study).

The results were plotted to check for correlation, and the coefficient of determination (R^2^) and correlation coefficient were calculated, where applicable. Statistical significance for the correlation coefficient was also checked afterwards using the Shapiro–Welch *t*-test. This comparison was not made for the whole length of the simulation (sowing until harvesting), just until the crop’s senescence, which for winter wheat in Hungary is around the beginning of June. This length was chosen because the chlorophyl concentration diminishes during senescence [39], lowering the NDVI value, while the biomass and CC still present a growing trend.

## 3. Results

The model performance was evaluated through comparison with NDVI-based biomass and crop cover estimates. This was done for winter wheat for the years of 2020 and 2021 at the experimental site in Gödöllő. One important difference from both years is that in 2021 there were more available dates to calculate NDVI due to the weather conditions in the region. Because of that, in 2021, the comparison was done until the beginning of senescence, and for 2020, the last suitable-quality satellite data were available for the beginning of May.

**Figure 4:**
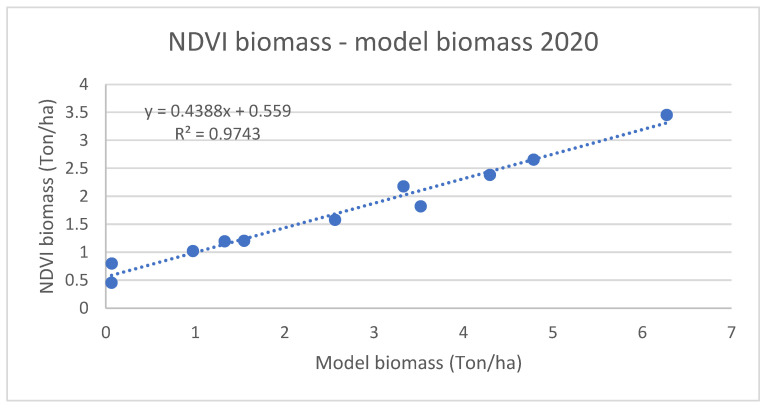
Comparison between NDVI biomass and modeled biomass for winter wheat in 2020.

**Figure 5:**
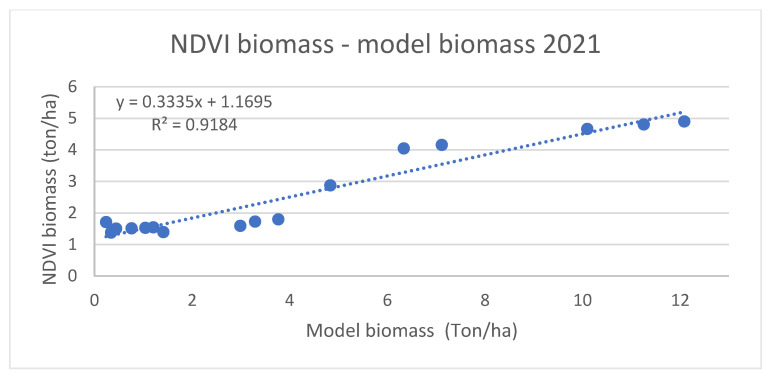
Comparison between NDVI biomass and modeled biomass for winter wheat in 2021.

Figure 4 and Figure 5 present NDVI-based biomass estimates compared with model estimations for 2020 and 2021, respectively, both indicating high correlation.

**Figure 6:**
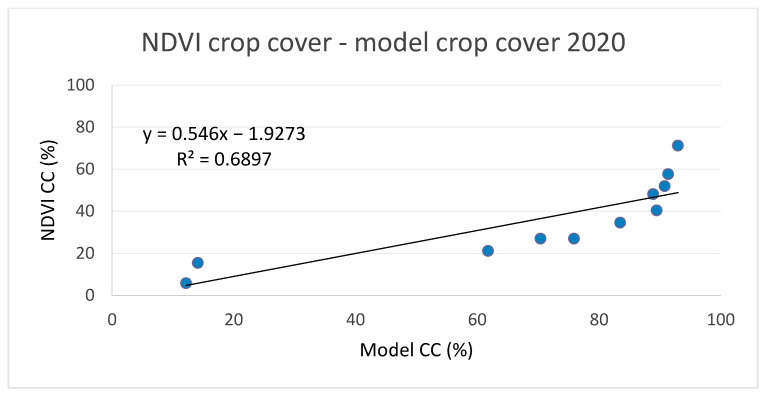
Comparison between NDVI canopy cover and modeled canopy cover for winter wheat in 2020.

**Figure 7:**
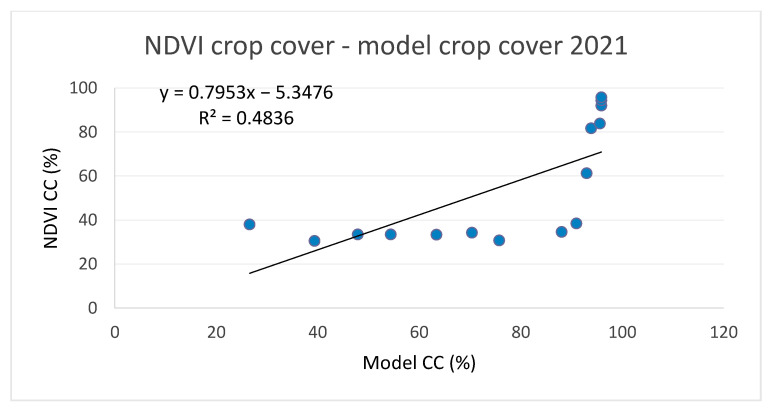
Comparison between NDVI canopy cover and modeled canopy cover for winter wheat in 2021.

**Figure 8:**
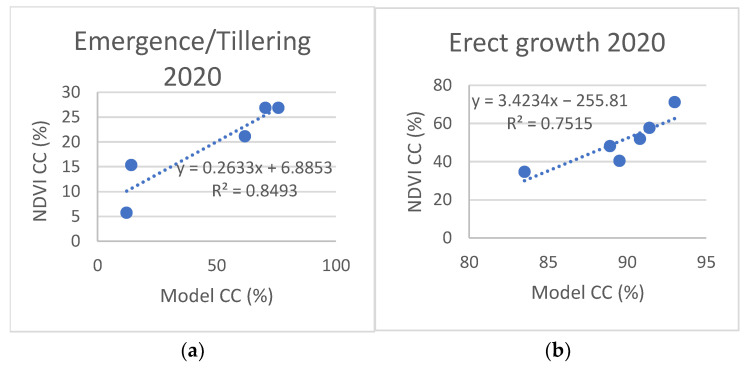
Comparison between NDVI and model-based canopy cover for emergence/tillering (**a**) and erect growth (**b**) of winter wheat, 2020.

**Figure 9:**
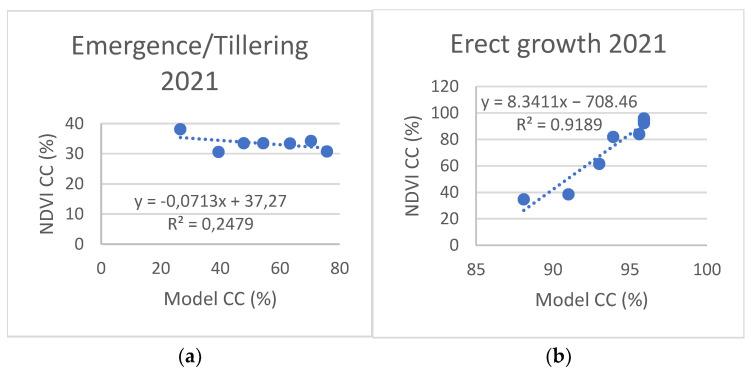
Comparison between NDVI and model-based canopy cover for emergence/tillering (**a**) and erect growth (**b**) of winter wheat, 2021.

NDVI-based crop cover (CC%) is compared with model estimates in Figure 6 and Figure 7. Due to the low correlation levels and a visible change in point distribution around the time of transition from tillering to erect growth (beginning of April), we also plotted values and calculated correlations for these two sections of the crop growth stage, as presented in Figure 8 and Figure 9.

The results show a very good linear correlation between modelled and NDVI-based biomass values, with R^2^ values of 0.97 and 0.91 for 2020 and 2021, respectively.

The comparison of canopy cover (CC%) presented significantly lower levels of correlation, with R^2^ values of 0.69 for 2020 and 0.48 for 2021. However, when the datasets were split up according to growth stages (Figure 8 and Figure 9), the R^2^ values significantly improved, particularly for the erect growth stages (Figure 8b and Figure 9b).

**Figure 10:**
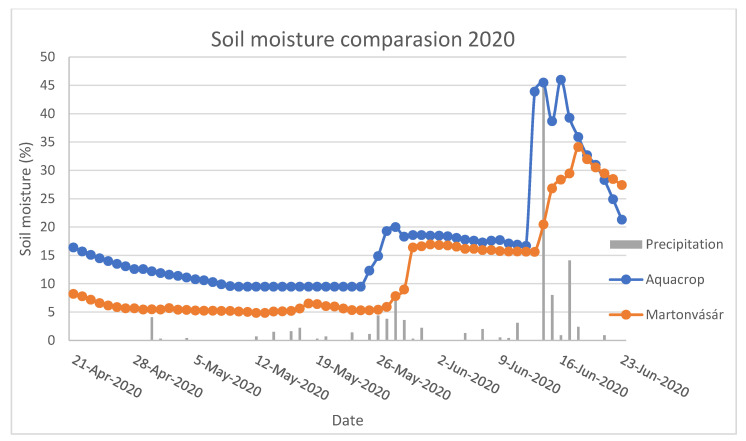
Comparison between modeled and measured soil moisture in Martonvásar for 2020.

Figure 10 presents the time series of modelled and measured soil moisture at the field in Martonvásár. The calculated correlation coefficient was 0.82, with an RMSE of 7.61. To check for statistical significance, we applied the Shapiro–Welch t-test; its result was a *p* value of 0.001, indicating statistical significance. It is possible to see as well that after two “significant” precipitation events, the values between the two timeseries started to change such that the model error decreased, but this decrease did not happen during the days on which the precipitation events occurred, where the difference between them was the highest.

**Figure 11:**
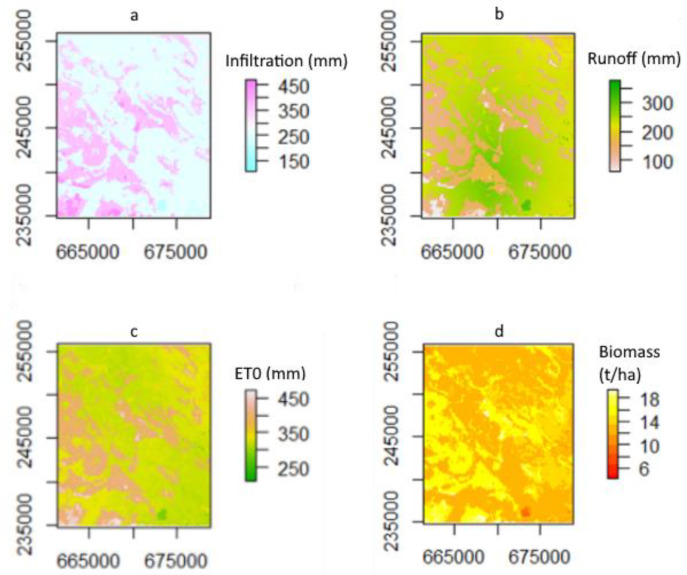
Spatial variation maps of seasonal infiltration (**a**), runoff (**b**), evapotranspiration (**c**), and biomass (**d**). (Projection: EPSG:23700.)

Figure 11 presents some of the spatial seasonal outputs (Figure 11a, infiltration; Figure 11b, runoff; Figure 11c, evapotranspiration; and Figure 11d, biomass) that the methodology can produce. The resulting maps clearly indicate the spatial heterogeneity of the area, showing that the different soil and climate properties were taken into consideration for the studied area.

Runoff and infiltration (Figure 11a,b) show some correlation between them, as is to be expected. When infiltration values are low, the runoff values in that region are higher, which clearly shows that the soil spatial variability for the area was taken into consideration.

**Figure 12:**
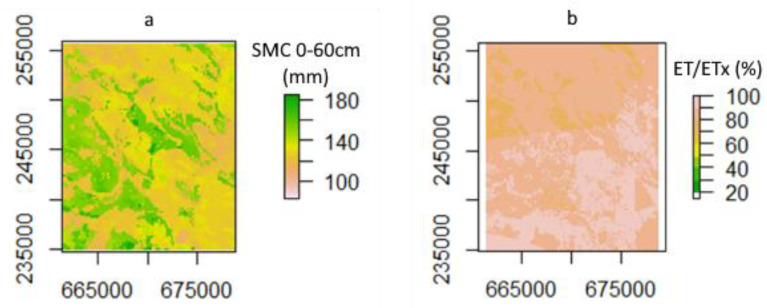
Spatial variation maps of daily water content for the rooting zone (**a**) and percentage of relative evapotranspiration (**b**) for 10 September 2020. (Projection: EPSG:23700.)

Figure 12 presents the spatial distribution of two selected variables on 10 September 2020, with the date selected arbitrarily, demonstrating the spatiotemporal information generated by the applied methodology. Figure 12a. presents the spatial variation in the water content for the rooting zone (0 to 60 cm), and Figure 12b. presents the percentage relative evapotranspiration (actual evapotranspiration divided by the maximum evapotranspiration) of the simulated crop in the area.

## 4. Discussion

From evaluating the soil moisture for maize in 2020 at Martonvásár (Figure 10), it is possible to see some trends between both of the timeseries, including a significant and good correlation coefficient between them. For the beginning of the timeseries, they showed a difference of around 7%, but that value decreased as two “significant” precipitation events occurred—one on 24 May and the other on 12 June. It is interesting to see that the values tended to get closer after the precipitation events. Another important point to take into consideration is how the AquaCrop model reacts faster to the rain in relation to what happens in reality in the field. This can be explained by how AquaCrop calculates soil moisture [40] and how it does not take into consideration some soil characteristics that would “smoothen” this rise in soil moisture after precipitation. Other factors (such as topography-related factors) are also not considered. Differences in actual crop characteristics, as well as potential errors in measurement, might also contribute to the difference.

One good conclusion that can be taken from this comparison is that the model works in a good manner when compared to data collected in the field, adding to the many different prior model validations [15,16,17,40].

We were able to demonstrate a good correlation between modeled and NDVI-based biomass until the senescence period of winter wheat for this region in Hungary. This correlation has been established with other crops as well in other different regions, but they might be region dependent [37,41]. Despite this, it is possible to say that AquaCrop can give good results for the modeled biomass, as they correlate quite well with the NDVI values for the area.

As for green canopy crop cover, the R^2^ values were lower than those for biomass when comparing NDVI and modelled values. However, the results seem to indicate a difference between how AquaCrop calculates CC values and how they are estimated from NDVI, particularly in the emergence/tillering stages of winter wheat. Lower correlation in these stages might also indicate specific differences in the model and actual field conditions, such as the occurrence/density of weeds within the field.

When looking at Figure 11 and Figure 12, the calculated seasonal results can be seen and are in line with what is expected; they show similar spatial variation, and values like infiltration and runoff have a correlation with one another, which is expected since both of those factors are dependent on the properties of the soil. However, in this scenario (also limited by the applied model), the effects of topography and surface conditions (such as ruggedness) have not been considered. According to Vereecken et al. [42] this approach can have benefits, too, since the very detailed local parameters are usually not representative enough for the whole modeled environment.

An advantage of the applied methodology is the potential generation of spatially distributed daily output data, which essentially allows the generation of “data cubes” for the specific study area, as demonstrated by Figure 12a,b. This opens the possibility for comparison and validation with Earth observation data, as well as for related agronomical applications, such as irrigation scheduling.

## 5. Conclusions

In general, we can conclude that the raster-based application of the AquaCrop model in an R-based environment was successful, with apparent and known limitations of the model still being present in the process. However, the methodology opens up new possibilities for spatial application, primarily depending on data availability. We also found that the modelled biomass values for winter wheat correlated well with NDVI-based estimates, while canopy cover estimations were more prone to additional effects, such as the effect of different growth stages of winter wheat on NDVI values. We believe that further application, testing, and fine-tuning of the methodology could be beneficial for a larger audience.

## Figures and Tables

**Table 1:** Input and output of major R functions within the SpatialAquaCrop package.

Function	Input	Output
Initial_AQC	If the model will run utilizing: field management, groundwater tables, or irrigation; If pre-determined crop files will be used or if they will be manually filled	Different .csv files depending on what the model will use to run and a data_fill.csv which has to be filled
Control_AQC	The filled data_fill.csv file; soil texture; saturated hydraulic conductivity; field capacity; wilting point; soil water content at saturation; precipitation; maximum temperature; minimum temperature; reference evapotranspiration	Different .csv files containing all the soil and climate data; a General Input text file that the AquaCrop plug-in uses to run the model
Spatial_AQC	The climate and soil .csv files	A .tif map of the seasonal and daily outpts; .csv files of the selected daily outputs
